# Why May COVID-19 Overwhelm Low-Income Countries Like Pakistan?

**DOI:** 10.1017/dmp.2020.329

**Published:** 2020-09-10

**Authors:** Inayat Ali, Shahbaz Ali

**Affiliations:** Department of Social and Cultural Anthropology, University of Vienna, Austria; Department of Anthropology, PMAS-Arid Agriculture University Rawalpindi, Pakistan

**Keywords:** COVID-19, health care, inequalities, low-income countries, Pakistan, poor governance, precarity, vulnerability

## Abstract

Since the coronavirus disease 2019, called COVID-19, has overwhelmed the high-income countries with ample resources and established health-care system, we argue that there are plausible concerns why it may devastate the low-income countries like Pakistan. Focusing on Pakistan, we highlight the underlying reasons, eg, demographic features, ineffective health-care system, economic and political inequalities, corruption, and socio-cultural characteristics, that create fertile grounds for COVID-19 to overwhelm low-income countries. This study presents Pakistan’s brief profile to demonstrate these underlying structures that may make low-income countries like Pakistan more vulnerable in the face of an unceasing COVID-19 pandemic. The study concludes that the country may make appropriate and possibly effective short-term preparedness measures to halt or slow the transmission of the virus, and deal with its current implications as well as it may pay significant attention to long-term measures to deal effectively with COVID-19’s longer-term effects. These measures will help them, including Pakistan, to deal appropriately with a similar future critical event.

During the past 6 mo, the effects of the ongoing coronavirus disease 2019, called COVID-19, that is caused by the severe acute respiratory syndrome coronavirus 2 (SARS-CoV-2), are visible and significant across the world. By the first week of July 2020, the virus has already infected more than 11 million people across the world, in which over 532,000 people have died.^1^ Shifting its epicenters from continents to continents, countries, both high and low income, have struggled to cope with its overwhelming impacts.

When high-income countries are overwhelmed, despite ample economic resources, including an efficient health-care system (services and providers), enough and critical concerns can be shown why the virus may have more devastating effects for low-income countries like Pakistan. Yet worries become more intense when there are fears about the second wave of the virus.^2^

Several underlying demographic factors, socio-cultural patterns, politico-economic factors, and elements related to the health-care system, which make these low-income countries extremely precarious to COVID-19 to have overwhelming impacts. There are visible forms of structured inequalities and institutionalized violence that are particularly a result of politico-economic structures. Profiles of these countries are very similar to each other: inappropriate health-care systems (lacking health-care services and providers); food scarcity, including malnutrition and stunting; poor and corrupt governance; low-economic resources; overwhelmed demographic characteristics and distinguishable socio-cultural patterns (eg, sharing the same spaces to sleep); significant prevalence of communicable and noncommunicable diseases. All these factors substantially contribute to weak systems, including immune and health systems.

There is usually an interplay among social, economic, and political factors that underpin health emergencies and positively shape the course of health interventions.^3^ Hence, a complex interface between pathogen biology and human, vector, and reservoir behavior is undeniable.^4^ The causal assemblages and contextualized epidemiologically notable behaviors in socio-cultural and politico-economic systems eventually contribute to systematic forms of disparities as well as either effective or ineffective structural interventions.

Focusing on Pakistan, we highlight those existing forms of institutionalized inequalities and vulnerabilities—associated with its demographic characteristics, socio-cultural factors, health-care-related elements, and politico-economic structures—that make the country stand on the verge of devastation in the face of a challenging situation, including the ongoing pandemic. When cases of the virus are decreasing in many countries, the virus is significantly spreading in Pakistan. After reporting the first infection on February 26, 2020, as of July 06, 2020, over 231,000 people have been infected in Pakistan, including over 4700 deaths.^1^ When many countries, especially in Europe, have eased the month-old precautionary measures due to decline in cases, Pakistan, like many South Asian countries, seems amid the peak and with overwhelming challenges. In the following section, we briefly revisit the above-mentioned characteristics: our argument may be seen against the backdrop of these critical features.

## DEMOGRAPHIC CHARACTERISTICS

Globally, Pakistan is the fifth most populous country: the total population is approximately 212.82 million.^5^ According to *the Pakistan Demographic and Health Survey 2017-18*, around 10% (of the sample size) of households lack water, soap, or other appropriate cleaning agents in place for handwashing.^6^ * Around 30% have no appropriate sanitation facility that is not shared with other households,^†^ and merely 25% have flush toilets linked to a septic tank.^6^ Fifty percent of women have no education compared with 34% of men, and net (school) attendance ratio (NAR) is 59% at the primary level and 38% at the middle/secondary level.^6^

## HEALTH-CARE SYSTEM: SERVICES, PROVIDERS, AND DISEASES

According to the *Economic Survey of Pakistan 2018-19*, Pakistan has approximately 1300 public sector hospitals, 5530 Basic Health Units (BHUs), 700 Rural Health Centers (RHCs), and 5680 dispensaries.^5^ The following was the ratios: 1 doctor per around 970 people, 1 dentist for around 9450, and 1 hospital bed for 1610 people.^5^ In terms of the provision of health care, rural and urban areas of the country report a large difference. Urban areas have enough and more proper facilities than rural populations.

In addition, several infectious diseases are prevailing in the country: eg, HIV/AIDs, hepatitis, measles, and polio. Witnessing around 8 outbreaks of HIV since 2003, Pakistan has been declared the second-fastest HIV growing country across Asia.^7^ Approximately 80% of the world’s hepatitis patients live in Egypt and Pakistan: of which approximately 18 million people have been infected with hepatitis in Pakistan.^8,9^ Pakistan is in the top 10 countries for the highest absolute number of neglected tropical diseases (NTDs) infections.^10^ In 2013, one study found that around 80 million individuals suffered from 1 or more chronic conditions, such as cardiovascular diseases, cancers, respiratory diseases, diabetes, and mental disorders.^11^ Measles has infected around 7000 children in 2019, and it caused a severe outbreak in 2012-13 infecting thousands of deaths and causing hundreds of deaths.^12,13^ Besides, Afghanistan, poliovirus is still prevalent in Pakistan.^12^

Moreover, malnutrition in the country is a great concern: 38% of children are “stunted” (short for their age), 7% are “wasted” (thin for their height), and 3% are overweight (heavy for their height).^6^ The next most vulnerable group for malnutrition is women.^14,15^ The percentage of malnutrition, food insecurity, and hunger increases in rural areas, especially in Thar Desert of Sindh province.

## ECONOMIC INEQUALITIES AND PERSISTENT CORRUPTION

Economic disparities are prominent in Pakistan. Around 25% of the country’s population lives below the poverty line (earning the US$2 per day).^5^ For gender and geographical area, these differences further intensify. The female population and people settled in rural areas fall into the lowest wealth quantiles.^6^ For gender related-inequalities, Pakistan was at 133 on the Gender Inequality Index (GII) in 2017.^5^

Like many South Asian countries, widespread government corruption is continuing in the country: According to Transparency International in its Corruption Perception Index for 2019, the country stands at 120th out of 180 countries.^16^ This organization indicated an increase in corruption compared with 2018. Endemic corruption has impeded economic growth, ultimately leading toward a weaker country that cannot successfully face a challenge or emergency on its own.^17,18^

## SOCIO-CULTURAL FACTORS CONCERNING HEALTH CARE

A substantial number of people regard health and illness as an act of God. Keeping someone healthy, making someone ill, and recovering are believed to be predetermined.^19,20^ This perception may lead people to be less careful about preventive measures about COVID-19. During the ongoing pandemic, the media have repetitively reported that people are congregating, arranging marriages, and performing religious festivals.

Moreover, living together, especially in a joint and extended family in rural areas, is a highly valued cultural norm. Three and in some cases, 4 generations live together, sharing their spaces and coming into frequent body contact.^19,20^ Countrywide, the following average household was reported by a survey: urban 6.3 and rural 6.8.^6^ In rural areas, there are clusters of houses, locally called *Mohalla* or *Parro*, with 1 boundary wall.^19^ Each cluster may consist of around or over 100 individuals.

Many people, particularly living in rural areas, do not merely subsist on animals, they also share their living spaces with them.^19^ Economically marginal people often share spaces with their cattle, sleeping in the same place at night. These people do not consider cattle, including their feces, as unhygienic or harmful to health. They dry dung and use it as a fuel for cooking food. It is primarily landless or small landowners in rural areas who significantly subsist on livestock.^21^ According to 1 report, livestock is considered as a “secure” source of livelihood for the people of Thar.^22^ An often-used expression is, “Thar depends upon animals, not crops,” and another expression reads, “a poor man [person] was 1 who owned no cattle or goat.”^23^ These expressions illuminate the importance of animals for the inhabitants and the human-animal relationship. In contrast, studies have revealed about the transmission of vectors from animals to humans.^24^ Annually, zoonoses cause morbidity in billions and mortality in millions in humans.^25^ Due to this effect, zoonotic diseases have become a significant public health concern, eg, COVID-19.^26^ Nevertheless, these animal-dependent people have no or little choice about their level of proximity with their animals.

Furthermore, like handshaking and hugging, eating with hands are appropriate cultural norms, in part due to cultural mores but also to the fact that about 25% of the population lives under the poverty line and cannot afford the required cutlery. Not engaging in these behaviors is normatively regarded as highly inappropriate and unethical. Of course, these cultural norms also supply vectors for disease transmission, and they are changing in response.

Several rumors and conspiracy theories prevail in the country, particularly about vaccination programs.^4,13,19,27^ There are several speculations about the “hidden” interests of vaccination campaigns, eg, a “Western” plot to sterilize Muslim women, and potential “side-effects” of vaccines that may kill children.^4,13,19,27^ Although suspicion about vaccination has not spilled over into suspicion about COVID-19, thus far, there are various competing narratives with unknown and untraceable sources, which have emerged, specifically in the villages (Salma, Ali and Ali, unpublished). Stories have surfaced to trace a “hidden” agent: the pandemic is “bioengineered” either by the United States or by “Big Pharma.” Speculations also contain home remedies, such as drinking garlic water (which might help), or “blowing hot air from a hairdryer through your nostrils,”^28^ or “killing” the infected people, “shaving one’s head,” and the “miraculous” birth of an infant.^13,19,29-31^

## GOVERNMENT RESPONSE TO THE COVID-19 PANDEMIC

To contain this virus, Pakistan has implemented several measures. After cases of COVID-19 in China, Pakistan suspended flights to China, then to Iran, Qatar, and Italy.^28^ In the beginning, the country sent the specimen to China and the United States due to the unavailability of test kits in the country, and later on, imported 1000 kits from China.^28^ On March 13, when the virus infected around 30 people, the government closed educational institutions, shut the border with Afghanistan and Iran, and opened a quarantine camp at Pak-Iran border.^28,31,32^ Afterward, the country banned congregations, including conferences and gatherings.^28,31,32^ On March 17, the country’s Prime Minister ruled out the option of lockdown while considering that 97% of patients recover.^33^ Nonetheless, Sindh province already implemented the lockdown. Later, the country went into lockdown and opened more quarantine centers.^28^ Police and army were being deployed across the country, including booking the “violators” under Section 188 of the Pakistan Penal Code: the penalty includes 6 mo in prison or fine or both.^28^

Furthermore, the government also created a Corona Relief Tiger Force to make people aware of the severity of COVID-19 and the importance of following standard operating procedures (SOPs).^28^ Although the government has distributed food items among daily wage laborers, people have criticized this distribution not just because of the number but also due to being photographed (with selfies) and shared on social media.

On May 9, 2020, the government lifted the countrywide lockdown. The Prime Minister argued that the “elites” want to extend the lockdown as they have “spacious homes” and sufficient income; thus, they would not be affected by the fallout of the measure.^29^

Given that, while considering it as the “pioneering” initiative, the country has changed lockdown into a “smart” lockdown.^34,35^ By that, the government means to place virus hotspots under lockdown.^28^ For instance, the “15-d smart lockdown” ended in 7 cities of Punjab province while this lockdown has been extended in many parts of Lahore city. Under this lockdown, educational and training institutions, restaurants (except take away), marriage halls, cinemas, business centers will remain shut. Sporting, social, and religious events are also banned.

## A WAY OF CONCLUSION

By the time we are writing this artice (July 2020), the virus is steadily spreading in Pakistan, and has already overwhelmed the country due to its critical state of socio-cultural, economic, and political systems. The above-discussed forms of inequalities—related to demographic landscape, economic patterns, health-care system, political landscape, and socio-cultural features—characterize it as a vulnerable county to COVID-19, including other similar overwhelming events that require a prompt and resourceful response.

The virus has not spared high-income countries from significant effects; hence, dealing inappropriately with it may overwhelm low-income countries like Pakistan. The virus may exert unbearable consequences on Pakistan’s socio-cultural patterns, economy, and health-care system, including the emotional and mental health of people.

It is, therefore, suggested that these low-income countries may make short- as well as long-term measures in advance. Short-term, the country may take appropriate and possibly effective measures to halt or slow the transmission of the virus and minimize its overwhelming effects at an individual and institutional level. They may learn lessons from high-income countries with effective health-care systems, low-population, good hygienic practices, and different socio-cultural patterns, which have substantially suffered.

Long-term measures may include devising and implementing those policies, which minimize the rising disparities and inequalities. Specifically for Pakistan, it is important to bring that 25% of the total population above the poverty line, so that they may not always stay on the verge of danger. The efforts may address the growing sociocultural, economic, and political inequalities. Among other probable solutions, the focus may be on the quantity and quality of education, as well as health, infrastructure, and employment. Yet rural areas need considerable attention with regard to it, because the difference between rural and urban areas should be minimized.

## Footnotes

* While following stratified 2-stage sample design. First, 580 clusters were selected, and in the second stage, which involved systematic sampling, a fixed number of 28 households per cluster was chosen—the total sample size was approximately 16,240 households.

† It includes “any non-shared toilet of the following types: flush/pour flush toilets to piped sewer systems, septic tanks, and pit latrines; ventilated improved pit (VIP) latrines; pit latrines with slabs.”^5^
